# A Life Prediction Model Considering Material Ductility in Multiaxial Fatigue Damage Analysis

**DOI:** 10.3390/ma18071597

**Published:** 2025-04-01

**Authors:** Xiaoting Liu, Xuding Song, Yuanzhe Dong, Wanjin Guo

**Affiliations:** 1 Key Laboratory of Road Construction Technology and Equipment, Ministry of Education, Chang’an University, Xi’an 710064, China; 2 EFORT Intelligent Equipment Co., Ltd., Wuhu 241060, China

**Keywords:** multiaxial fatigue, life prediction, ductility, virtual shear strain energy density, critical plane-energy criteria

## Abstract

In this paper, a novel multiaxial fatigue damage parameter was developed based on the critical plane-energy method. The proposed damage parameter considers the sensitivity difference of the material ductility to the out-of-phase loads and takes the function of material elongation as an adjustment function. At the same time, the mutual promotion of shear stress and normal stress in the process of microcrack propagation was considered to characterize the friction and interlock phenomenon on the crack surface in the new parameter. It is indicated that the new model has reliable prediction results for all verification materials. Furthermore, the new model also has the benefit of not requiring the introduction of a new material constant.

## 1. Introduction

Multiaxial loads are used for many structural components in engineering, such as airframes and turbines [1,2,3], making the study of multiaxial fatigue extremely important [4]. Multiaxial fatigue is a practical and challenging problem. Despite extensive research on this topic worldwide, there is still a lack of more reliable criteria for multiaxial fatigue due to its complexity and time-consuming and expensive experimental studies [5].

**Figure 1:**
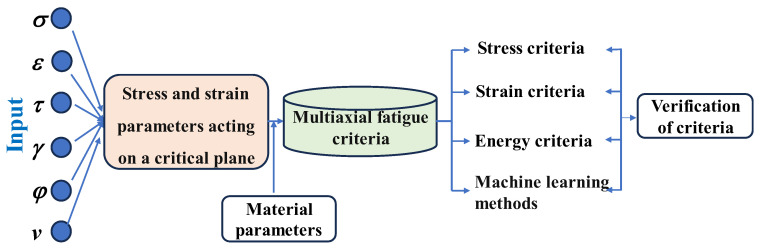
The flowchart of multiaxial fatigue life prediction.

The establishment and evaluation of the multiaxial fatigue criteria have attracted a large number of scholars [6,7]. Four main criteria can be used to classify multiaxial fatigue life prediction methods—stress-based criterion, strain-based criterion, energy-based criterion [8], and machine learning methods [9,10]—as shown in Figure 1. The critical plane criterion is the most widely accepted fatigue theory among them because the experimental results show that in the presence of multiaxial fatigue, both the beginning and the growth of cracks in the materials occur on a specific plane, called the critical plane, rather than randomly [11]. Once the critical plane has been identified, different stresses and strains on the critical plane can be combined in accordance with specific rules to develop multiaxial fatigue life models, such as the maximum shear strain model (MSSM) [12], WB criterion [13], KBM criterion [14] based on the maximum strain criterion, the most popular FS criterion [15], and the stress-based Findley criterion [16]. The expressions for these fatigue criteria are shown in Table 1. In recent years, the effectiveness of data-driven life prediction models has been empirically verified in different engineering fields [17]. However, machine learning-based methods are expensive to apply in practice due to the lack of data.

The greater life prediction capabilities of the Fatemi–Socie (FS) critical plane model make it more popular than others. The primary reason for crack initiation is thought to be cyclic loading in this model, and the view that the normal stress on the maximum shear strain plane affects the friction and interlocking of the crack surface is consistent with the experimental phenomenon. Furthermore, the FS model states that non-proportional hardening reduces fatigue life. However, to account for the impact of normal stress on crack growth, the FS criterion introduced the material constant *k*. Fitting *k* is cumbersome, particularly when test data are limited, and the additional material constant is determined using various approaches, which frequently yields inconsistent life prediction outcomes [18].

Although these damage indicators based on stress and strain alone are widely used, they cannot reasonably express the materials’ constitutive behavior, resulting in unsatisfactory prediction results. However, even though the life prediction criterion based on energy may describe the constitutive behavior of materials, energy is a scalar and does not adequately express the damage mechanism; for this reason, it has drawn criticism. Accordingly, the multiaxial criterion of the energy method considering the theory of the critical plane is further proposed [19,20].

As shown in Table 1, the SWT method, which is the earliest critical plane-energy criterion, is based on the influence of the principal strain range and the maximum stress acting on the critical plane. Because only the normal strain energy is taken into account, this method is typically more accurate in predicting life under uniaxial fatigue load conditions [20]. Liu believed that the damage of materials under multiaxial loadings includes two components—normal strain energy and shear strain energy—and developed the Liu I criterion and the Liu II criterion for different material failure mechanisms, which are suitable for type I failure modes and type II failure modes, respectively [21]. It is important to note that not all normal strain energy will result in damage, since crack closure effects on the surface of the crack may occur [22]. This leads to multiaxial failure of materials, with different weights assigned to the shear strain energy and normal strain energy components. In addition, it is well known that negative average stress increases fatigue life, while positive average stress decreases fatigue life [23]. The Varvani criterion considered the difference in damage between the normal strain energy and shear strain energy on the critical plane by dimensionless treatment of the two sections [24]. Chen et al. criticized and improved the SWT model, known as the CXH model, which took into account both the normal strain energy and the shear strain energy acting on the critical plane and assumed that normal and shear work have the same weight [25]. The CXH model has been demonstrated to have good predictive performance, particularly in ductile materials. However, the effect of average stress was ignored in the CXH model [8]. Although some scholars believe that the material ductility leads to differences in the fatigue life under proportional and non-proportional loads, few life prediction models have taken into account the material ductility parameters so far.

**Table 1:** Multiaxial fatigue criteria.

	Criteria	Expression	Refs.
Stress	Findley	${\left(\tau_{a}+k_{F}\sigma_{n}\right)}_{\max}=f\left(2N_{f}\right)$	[16]
Strain	MSSM	$\frac{\Delta \gamma_{\max}}{2}=f\left(2N_{f}\right)$	[12]
	KBM	$\frac{\Delta \gamma_{\max}}{2}+k_{BM}\cdot \Delta \varepsilon_{n}=f\left(2N_{f}\right)$	[14]
	WB	$\frac{\gamma_{\max}}{2}+k_{WB}\cdot \frac{\Delta \varepsilon_{n}}{2}=f(2N_{f})$	[13]
	FS	$\frac{\Delta \gamma_{\max}}{2}\left(1++k\frac{\sigma_{n,\max}}{\sigma_{y}}\right)=f\left(2N_{f}\right)$	[15]
Energy	SWT	$\frac{\Delta \varepsilon }{2}\sigma_{n,\max}=f\left(2N_{f}\right)$	[20]
	Liu I	$\Delta \tau \Delta \gamma +{\left(\Delta \sigma_{n}\Delta \varepsilon_{n}\right)}_{\max}=f\left(2N_{f}\right)$	[21]
	Liu II	${\left(\Delta \tau \Delta \gamma \right)}_{\max}+\Delta \sigma_{n}\Delta \varepsilon_{n}=f\left(2N_{f}\right)$	[21]
	Varvani	$\frac{\Delta \sigma_{n}\Delta \varepsilon_{n}}{\sigma_{f}^{\prime }\varepsilon_{f}^{\prime }}+\frac{\Delta \tau_{\max}\Delta \gamma_{\max}}{2\tau_{f}^{\prime }\gamma_{f}^{\prime }}=f\left(2N_{f}\right)$	[24]
	CHX	$\Delta \gamma_{\max}\Delta \tau +\Delta \varepsilon_{n}\Delta_{n}=f\left(2N_{f}\right)$	[25]

*k_F_*, *k_WB_*, and *k_BM_* are material parameters.

The purpose of this article is to present a balanced multiaxial fatigue damage parameter based on the multiaxial fatigue test results of AA2024-T351. This parameter defines the plane where the maximum shear strain amplitude is located as the critical plane, and the virtual shear strain energy density as the primary control parameter. The combination of ductility parameters and non-proportional load parameters as adjustment parameters and the promotion effect of normal stress and shear stress on the crack surface is also considered. First of all, the Basquin–Manson–Coffin equation was used to correlate damage parameters to construct a life prediction model. Next, the new model was contrasted with the MSSM, SWT, and FS models. The robustness and accuracy of the new model were methodically confirmed using the experimental results of AA2024-T351 and the other five checked materials from the works of literature subjected to multiaxial fatigue loading situations. In addition, the advantage of the new model was that no additional material constant was introduced.

## 2. Experimental Tests and Results

### 2.1. Uniaxial Fatigue Experiment

**Figure 2:**
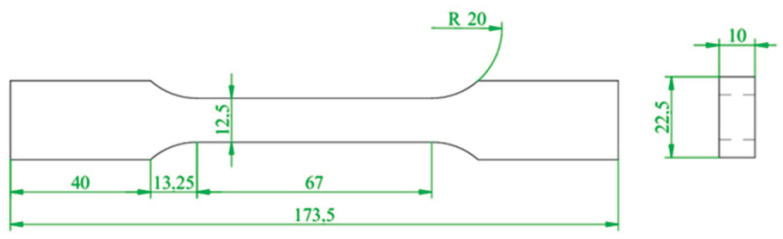
Schematic diagram of the monotonic tensile specimens (unit: mm).

**Figure 3:**
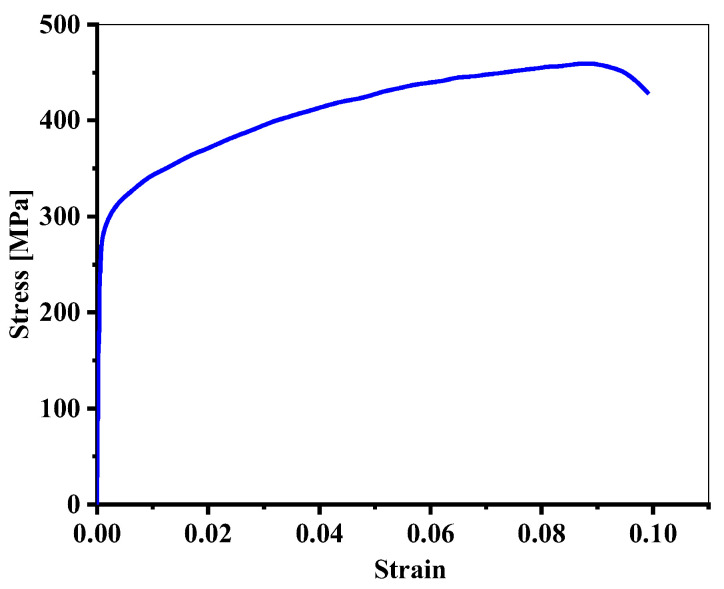
The monotonic tensile curve of AA2024-T351.

**Figure 4:**
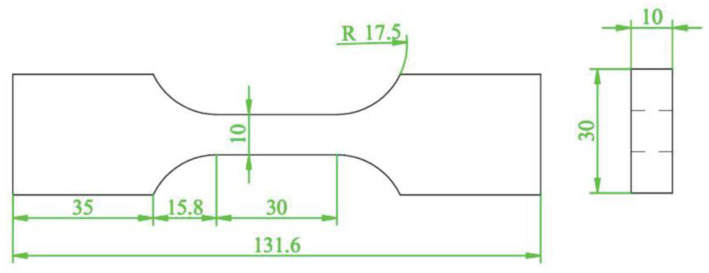
Schematic diagram of the uniaxial fatigue specimen (unit: mm).

Firstly, AA2024-T351 was taken as the research object. The test was conducted using an Instron 8801 testing system to perform a monotonic tensile test with a displacement control method and a tensile rate of 0.5 mm/min. According to GB/T 228.1-2021 [26], a dumbbell specimen was used to test the mechanical properties of the AA2024-T351, as shown in Figure 2. The monotonic tensile curve is shown in Figure 3, and its tensile properties are shown in Table 2. In order to obtain the fatigue parameters of AA2024-T351, according to ASTM E606 [27], the strain-controlled low-cycle fatigue test was carried out on the non-notched dumbbell specimens with the shape and size of the specimen as shown in Figure 4. The strain amplitude was 0.4%, 0.5%, 0.6%, 0.7%, 0.8%, 0.9%, and 1.0%, and the strain amplitude ratio Rε = −1 with frequency *f* = 1 Hz. The test was not stopped until the sample fractured.

**Figure 5:**
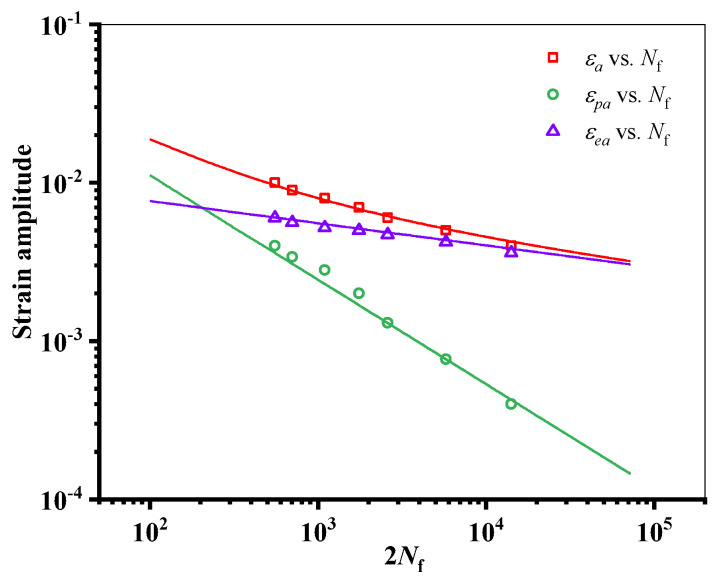
Uniaxial fatigue strain–life curve of AA2024-T351.

Figure 5 is the uniaxial fatigue test results of AA2024-T351. The Manson–Coffin formula (as Equation (1)) was used to fit the test data. The strain–life curve obtained is shown in Figure 5, and the material fatigue parameters are shown in Table 3.

(1) $$\varepsilon_{a}=\varepsilon_{ea}+\varepsilon_{pa}=\frac{\sigma_{f}^{\prime }}{E}{\left(2N_{f}\right)}^{b}+\varepsilon_{f}^{\prime }{\left(2N_{f}\right)}^{c}$$

In Equation (1), *ε_a_* is the total strain amplitude, *ε_ea_* is the elastic strain amplitude, *ε_pa_* is the plastic strain amplitude, and $\sigma_{f}^{\prime }$ and $\varepsilon_{f}^{\prime }$ are the fatigue strength coefficient and fatigue ductility coefficient, respectively. *b* and *c* are the fatigue strength and ductility exponent, respectively.

### 2.2. Multiaxial Fatigue Test

**Figure 6:**
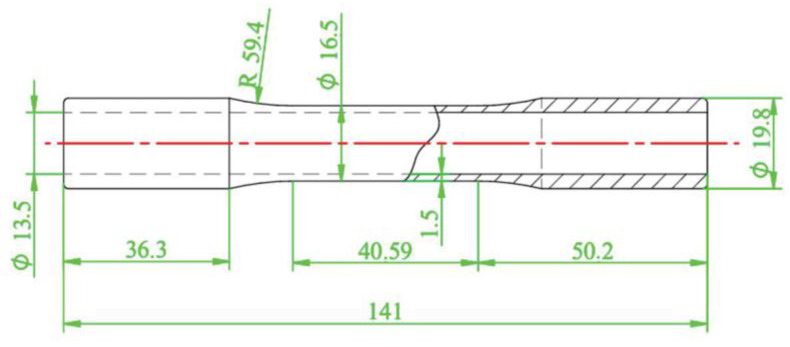
Schematic diagram of thin-walled round tube specimen (unit: mm).

Our team conducted a multiaxial fatigue test for AA2024-T351, and the test details fully described in Ref. [28] are briefly summarized here. A thin-walled round tube specimen with a wall thickness of 1.5 mm and an external diameter of 19.8 mm was selected. A uniform surface roughness of 0.2 μm was ensured on the inner and outer surfaces of the specimen to eliminate the influence of machining marks, as shown in Figure 6. All multiaxial strain-controlled fatigue tests were performed at room temperature on a closed-loop servo-hydraulic tensile–torsional testing system, and fatigue tests on tubular specimens were loaded in both axial and torsional directions. The details of the experimental design are shown in Table 4. A triangular waveform that controls the axial strain and torsional strain applied by the load, with a strain ratio equal to −1.0 and a loading frequency of 1 Hz. The strain paths used in this study are in-phase and 90° out-of-phase loading. Each test was performed at equal von Mises strain amplitude values with strain amplitude ratios of 1.73 and 1.24.

The measured values of AA2024-T351 multiaxial fatigue life under different loading paths are shown in Table 3. When the equivalent effect value is 0.0055 and the strain amplitude ratio is 1.73, the average lifetime of the specimen under proportional and non-proportional loads is 7229 cycles and 11,191 cycles, respectively. In a similar case, when the strain amplitude ratio is 1.24, the lifetime of the specimen is 6905 cycles under proportional load, while the average lifetime under non-proportional load is 12,175 cycles. It can be concluded that under the same conditions, the life of AA2024-T351 under proportional load is shorter than that under non-proportional load.

## 3. Effect of Materials’ Ductility on Multiaxial Fatigue Life

Generally speaking, it is acknowledged that the reduction in fatigue life under out-of-phase loads compared to in-phase loads is caused by multiaxial cyclic hardening. For example, compared to proportional loads, non-proportional loads drastically shorten the fatigue life of ductile materials like wrought aluminum and forged or unwelded steel [29]. Therefore, damage resulting from non-proportional hardening is frequently taken into account by life estimation methods, resulting in accurate predictions. However, in Section 2, the lifetime of AA2024-T351 under non-proportional load is greater than that under proportional load. Also, it has been reported that fatigue life increases under multiaxial non-proportional loads in certain situations; for example, cast magnesium and aluminum alloys with low elongation showed a significantly longer fatigue life under non-proportional loading [30]. As a result, the material’s ductility could influence the damage mechanism. According to reports [31], shear stress(strain) is the primary damage parameter in ductile materials, where the microcrack grows along the maximum shear plane after being nucleated along the slip system. When a crack nucleates in brittle materials, the microcrack grows along the crack surface, and the main damage parameter is normal stress or strain. The interaction between the normal stress and shear stress leads to damage to the semi-ductile material, so the damage parameter is a combination of normal stress(strain) and shear stress(strain). This implies that, as well as the non-proportional hardening parameter, the material’s ductility parameter may also be able to explain the damage.

**Figure 7:**
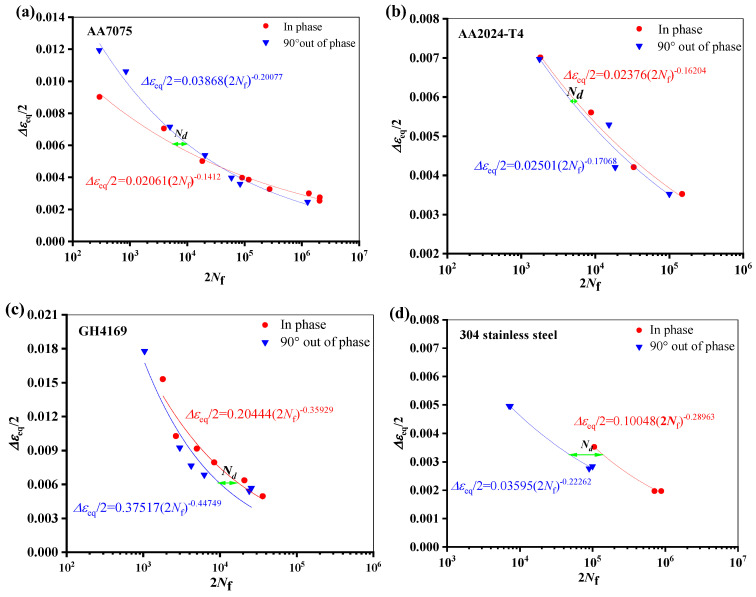
Effect of load paths on fatigue life at the same equivalent strain amplitude: (**a**) AA7075, (**b**) AA2024-T4, (**c**) GH4169, and (**d**) 304 stainless steel.

The fatigue life reduction of specimens from different materials under non-proportional multiaxial load is depicted in Figure 7. The results of tests on four materials’, namely, AA7075 [32], AA2024-T4 [33], GH4169 [34], and 304 stainless steel [35], are presented. The materials’ ductility parameters discussed in this article are derived from the corresponding articles and the material manual.

In Figure 4, the abscissa is twice the fatigue lifetime, the ordinate is the equivalence strain amplitude, and the variable *N_d_* represents the degree to which fatigue life under non-proportional loading is reduced in comparison to fatigue life under proportional loading. The expression is as follows: Equation (2) [36].

(2) $$N_{d}=\frac{N_{ipf}-N_{nonpf}}{N_{ipf}}$$

*N*_ipf_ represents the fatigue life under a proportional load, and *N*_nonpf_ represents the fatigue life under the same equivalent strain amplitude with a 90° non-proportional loading.

(3) $$\Delta \varepsilon_{eq}/2=\sqrt{{\left(\Delta \varepsilon /2\right)}^{2}+{\left(\Delta \gamma /2\right)}^{2}/3}$$

In Equation (3), Δε/2 is the axial strain amplitude, Δγ/2 is the shear strain amplitude, and, according to the von Mises criterion, Δε_eq_/2 is the equivalent strain amplitude.

**Figure 8:**
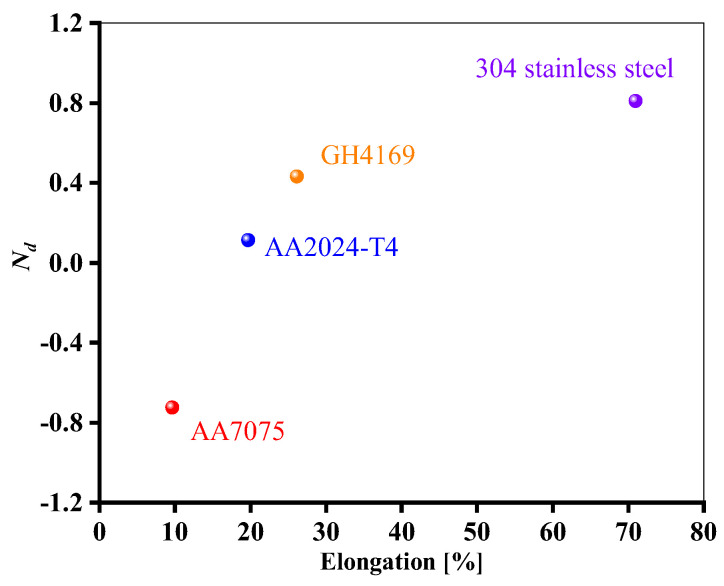
Effect of materials’ ductility on fatigue life under proportional and 90° non-proportional loadings (Δε_eq_/2 = 0.006).

**Figure 9:**
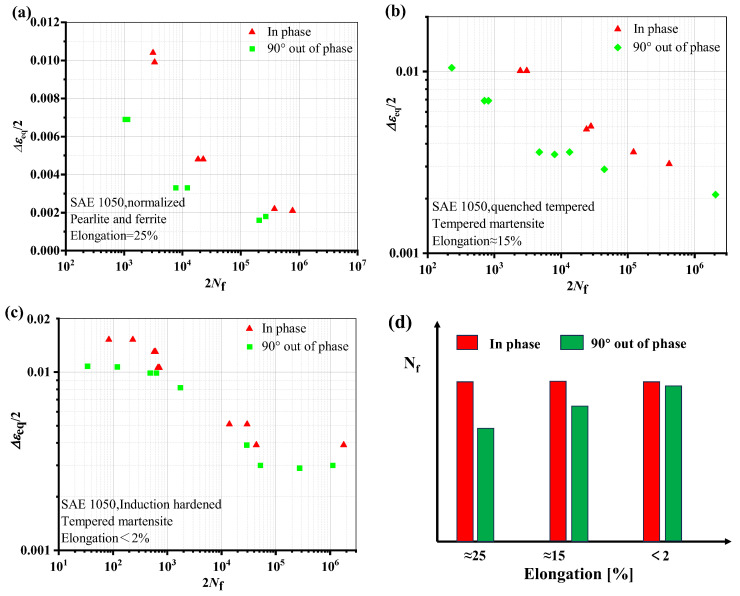
Effect of material ductility level on fatigue life for in-phase and 90° out-of-phase loads: (**a**) Elongation is 25%, (**b**) Elongation is 15%, (**c**) Elongation less than 2%, (**d**) Effect of elongation on life under non-proportional loads [30,37].

As seen from Figure 8, in the low cycle region, the life of AA7075 under non-proportional load increases, while the other three materials have a higher ductility level and a greater degree of life attenuation under non-proportional load. Similar cases were reported in the Refs. [29,30]. Figure 9 shows the results of strain-controlled multiaxial fatigue testing on SAE 1050 steel subjected to different quenching and tempering conditions. When the microstructure changes from bainite to more martensite, it is obvious that the material’s toughness is decreased. As can be seen from Figure 9d, the greater the ductility, the more obvious the life reduction under out-of-phase load. There is not much difference in fatigue life between proportional and non-proportional loading situations when the elongation is less than 2%.

In light of the results of the above analysis, it will be interesting to establish a relationship between the ductility parameter of the materials (i.e., elongation) and the multiaxial fatigue life.

## 4. Proposed Critical Plane-Energy Model

### 4.1. Establishing a Damage Parameter

Regarding the influence as mentioned above, the proposal for this parameter is based on the following three assumptions:

Firstly, the metals’ brittleness and ductility are not absolute, and it can reasonably be considered that the normal component and the shear component work together [8]. However, only a portion of the normal component causes damage because of the different degrees of crack closure effect on the critical plane [38]. Therefore, for the materials with shear failure characteristics, the virtual shear strain energy density at the critical plane is used as the primary index, and the maximum positive stress at the critical plane is used as a secondary index to express the damage the normal component has caused.

Secondly, microscopically, persistent slip bands (PSB) resulting from cyclic plastic deformation are commonly recognized as sources of crack initiation. Experiments have demonstrated that fatigue cracks usually initiate at the plane of the maximum shear strain and that persistent slip bands (PSB) occur very close to the direction of maximum shear strain under different load conditions [39]. Therefore, it has physical significance in determining that the critical plane is the one with the maximum shear strain amplitude.

Then, for most metal materials, the normal stress(strain) decreases as the shear stress(strain) increases, indicating that larger local deformation can overcome friction on the crack surface, and conversely, an increase in the normal stress(strain) can open up the crack surface and reduce the friction. According to Ref. [40], the damage parameter of the FS model can have good stability when *G*Δ*γ* is used as a normalized parameter, which explains that shear stress and normal stress are mutually promoted. In addition, Xu et al. developed a similar function that reflects the effect of the principal stress on fatigue crack propagation [41]. The formula is as follows:

(4) $$k^{\prime }=\frac{\sigma_{n,\max}}{4G\Delta \gamma }$$

where *σ*_n,max_ is the maximum normal stress on the critical plane, Δ*γ* and is the range of shear stress on the critical plane, and *G* is the shear modulus.

**Figure 10:**
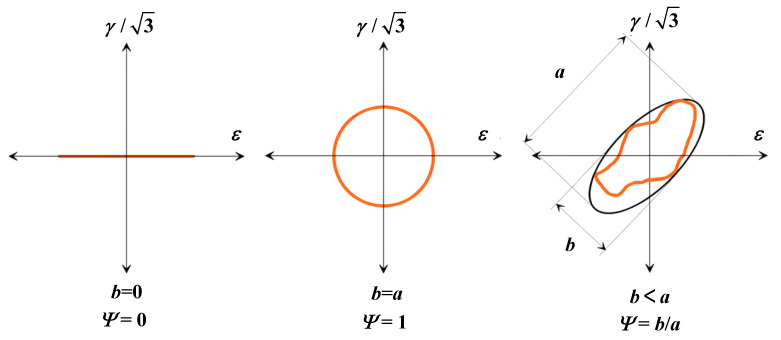
Load non-proportional parameter [42].

Finally, as the phase angle increases, the multiaxial fatigue life decreases. The results in Section 2 show that the higher the material’s ductility, the more sensitive the material is to out-of-phase loads. At the same time, the shear components of highly ductile materials under non-proportional multiaxial loads predominate, and the shear effect is more evident as the ductility level increases. In this study, load non-proportional parameter, *Ψ,* is determined from the ratio of the minor axis, b, to the major axis, a, of the minimum circumscribed ellipse defined by the strain path (Figure 10) [42]_,_ *δΨ* is used as the adjustment function under different loads at different materials.

The proposed damage parameter in this study was as shown in Equation (5):

(5) $$\begin{array}{l} \frac{\Delta \tau_{t}}{2}\frac{\Delta \gamma_{\max}}{2}\left[1+\delta \psi +\frac{\sigma_{n,\max}}{G\Delta \gamma }\right]=f(2N_{f}) \end{array}$$

where Δ*τ*_t_/2 is the amplitude of shear stress acting at the critical plane, Δ*γ*_max_/2 is the maximum shear strain amplitude acting at the critical plane, and *δ* is the ductility parameter of the material, i.e., elongation. *Ψ* is the non-proportional factor depending on the strain path.

### 4.2. Relationship Between Damage Parameter and Fatigue Life

**Figure 11:**
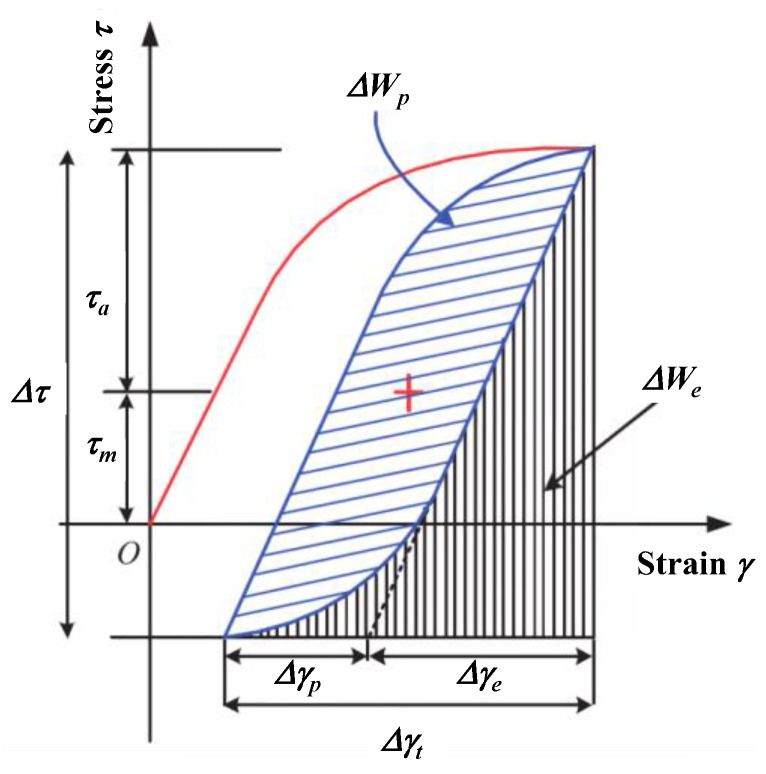
Cyclic strain energy for a uniaxial stress state [44].

Ellyin proposed cyclic strain energy density as a damage parameter that unifies high and low cycle fatigue by combining plastic strain energy density (Δ*W_p_*) and elastic strain energy density (Δ*W_e_*) that promotes crack initiation (Figure 11) [43]. The mathematical expressions for the fully reversed strain-controlled low-cycle fatigue results of the tests may be written as Equation (6):

(6) $$\frac{\Delta \gamma }{2}=\frac{\Delta \gamma_{p}}{2}+\frac{\Delta \gamma_{e}}{2}=\gamma_{f}^{\prime }{\left(2N_{f}\right)}^{c_{0}}+\frac{\tau_{f}^{\prime }}{G}{\left(2N_{f}\right)}^{b_{0}}$$

Furthermore, the best-fit line for controlled stress is given by Equation (7):

(7) $$\frac{\Delta \tau }{2}=G\frac{\Delta \gamma_{e}}{2}=\tau_{f}^{\prime }{\left(2N_{f}\right)}^{b_{0}}$$

The total strain energy expression is Equation (8)

(8) $$\frac{\Delta \tau }{2}\frac{\Delta \gamma }{2}=\frac{\tau_{f}^{\prime 2}}{G}{\left(2N_{f}\right)}^{2b_{0}}+\tau_{f}^{\prime }\gamma_{f}^{\prime }{\left(2N_{f}\right)}^{b_{0}+c_{0}}$$

Therefore, the relation between the fatigue life and the suggested new damage parameter is Equation (9):

(9) $$\frac{\Delta \tau_{t}}{2}\frac{\Delta \gamma_{\max}}{2}(1+\delta \psi +\frac{\sigma_{n,\max}}{G\Delta \gamma })=\frac{\tau_{f}^{\prime 2}}{G}{\left(2N_{f}\right)}^{2b_{0}}+\tau_{f}^{\prime }\gamma_{f}^{\prime }{\left(2N_{f}\right)}^{b_{0}+c_{0}}$$

## 5. Proposed Model Validation and Comparison

### 5.1. AA2024-T351

This section compared the life prediction performance of the new model with the popular FS model, MSSM, and SWT models under proportional and non-proportional loads. The shear parameters are obtained according to the von Mises equivalence criterion formula are shown in Equations (10)–(13) [45]:

(10) $$\tau_{f}^{\prime }=\frac{\sigma_{f}^{\prime }}{\sqrt{3}}$$

(11) $$\gamma_{f}^{\prime }=\sqrt{3}\varepsilon_{f}^{\prime }$$

(12) $$b_{0}=b$$

(13) $$c_{0}=c$$

**Figure 12:**
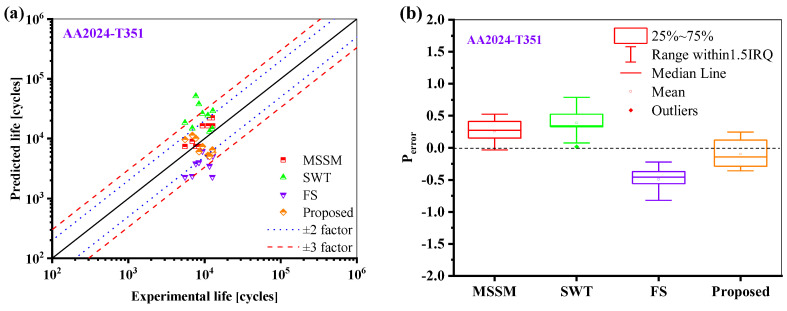
Comparison of the life prediction results of AA2024-T351 by different models: (**a**) Experimental life vs. Predicted life, and (**b**) box plot of predicted error.

Model prediction errors *P*_error_ have been evaluated by a probability analysis to show the differences between these methods visually [38]:

(14) $$P_{\mathrm{error}}=log_{10}(N_{fp})-log_{10}(N_{ft})$$

where *P*_error_ is the predicted error, *N*_fp_ is the predicted life, and *N*_ft_ is the true life. Block diagrams of prediction errors are created for different life prediction models. As can be seen from Figure 12, The prediction values of MSSM for AA2024-T351 are all within ±2 times the scattering band, but the overall prediction results are large. The life prediction results of the SWT model for AA2024-T351 are greater than the actual values, and the predicted values of the FS model for AA2024-T351 are lower than the experimental values. The prediction results of the proposed model are neither overestimated nor conservative, and most of them are within the ±2 times the scattering band.

### 5.2. Other Materials

**Figure 13:**
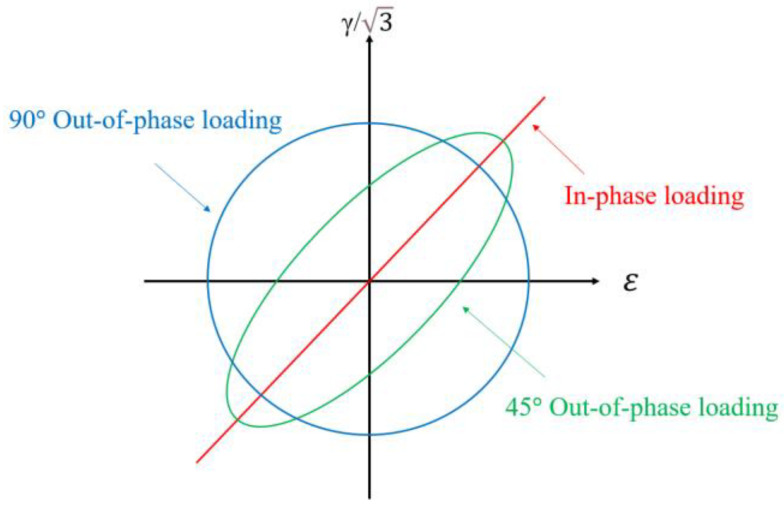
Multiaxial fatigue test load paths.

To confirm the precision and universality of the new model in other materials, five kinds of materials, namely, TC4 [12], GH4169 [34], 316LN [46], S460N [47], and pure Ti [48], were evaluated under different load paths. As depicted in Figure 13, the proportional loading path is symbolized by the straight red line, the green ellipse represents the 45° non-proportional load path, and the blue circle represents the 90° non-proportional load path.

The uniaxial fatigue parameters of checked materials can be seen in Table 5, and the specific details of the specimen are visible in the references. The ductility parameters of the five materials are given in Table 5 and can also be obtained by consulting the material manual.

**Figure 14:**
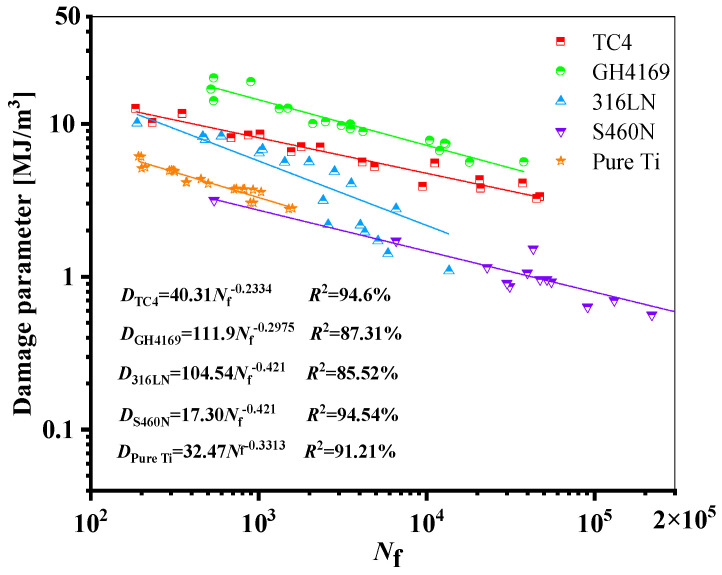
Damage parameters vs. fatigue failure cycles for the new model for five materials.

The relationship between the fatigue life of the five materials and the damage parameters under the proposed model is depicted in Figure 14. The relationship between the fatigue life and the damage parameters is fitted by the power-law function in a log-log scale, where the fatigue life is represented by the abscissa, and the equivalent damage parameter is defined by the left ordinate, which corresponds to proposed energy models, is the right ordinate. The results show that the damage parameters and life of the five materials have a high goodness of fit, i.e., *R*^2^ is greater than 85%, meaning that the damage parameters fall within a narrow scatter band of the curve.

**Figure 15:**
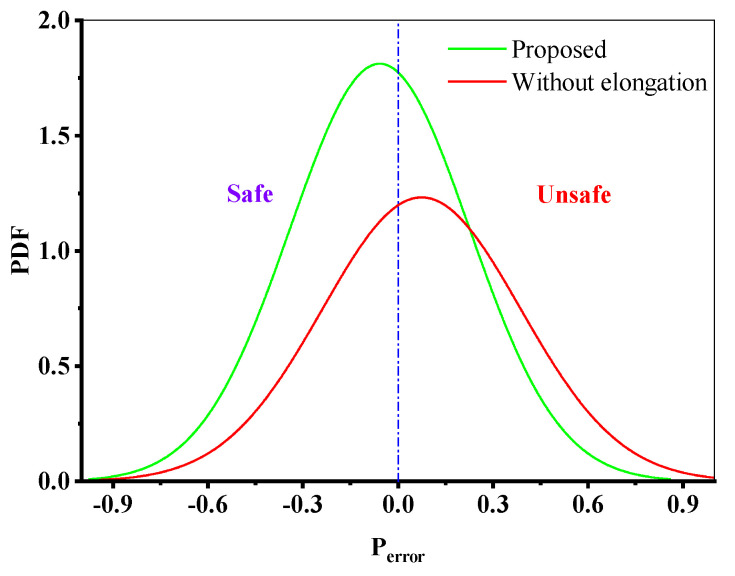
Effect of the ductility adjustment function of the materials on the *P*_error_ of the proposed model.

The impact of the ductility adjustment function *δΨ* on the prediction results of the proposed model is depicted in Figure 15, where it is evident that the presence of ductility adjustment parameters can raise the damage value, making the prediction results of the model more accurate and safer.

**Figure 16:**
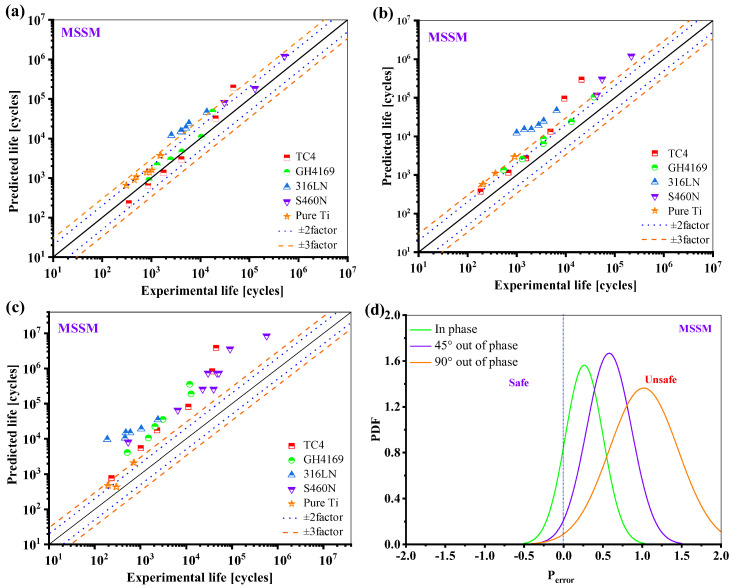
Prediction results of the MSSM: (**a**) in phase; (**b**) 45° out of phase; (**c**) 90° out of phase, and (**d**) the probability density function of the *P*_error_.

The prediction results of MSSM for the five materials are displayed in Figure 16. While the fatigue life under proportional loading is typically within ±3 times the scattering band, the results for the fatigue life prediction under 45° and 90° non-proportional loadings are overestimated.

**Figure 17:**
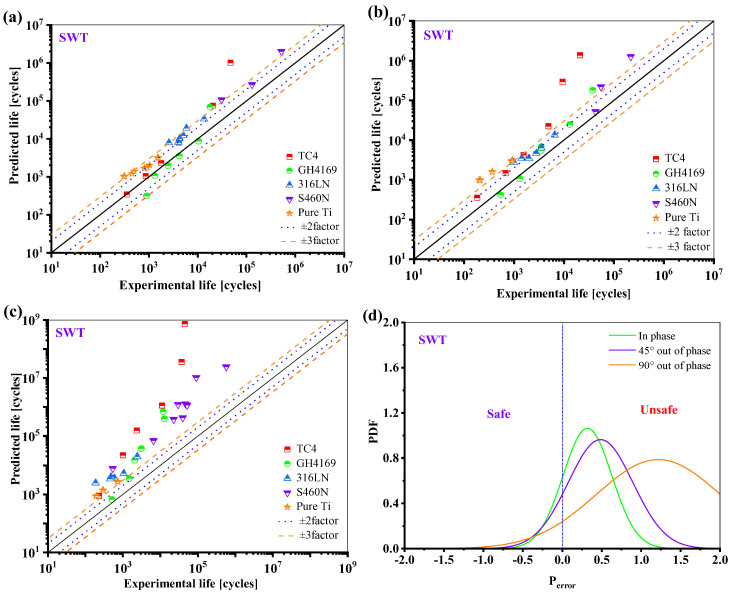
Prediction results of SWT model: (**a**) in phase; (**b**) 45° out of phase; (**c**) 90° out of phase, and (**d**) the probability density function of the *P*_error_.

Since the SWT damage parameter only considers the normal damage for shear failure materials, resulting in a very small damage value, the SWT model overestimates the lifetime predictions for all five materials, as shown in Figure 17.

**Figure 18:**
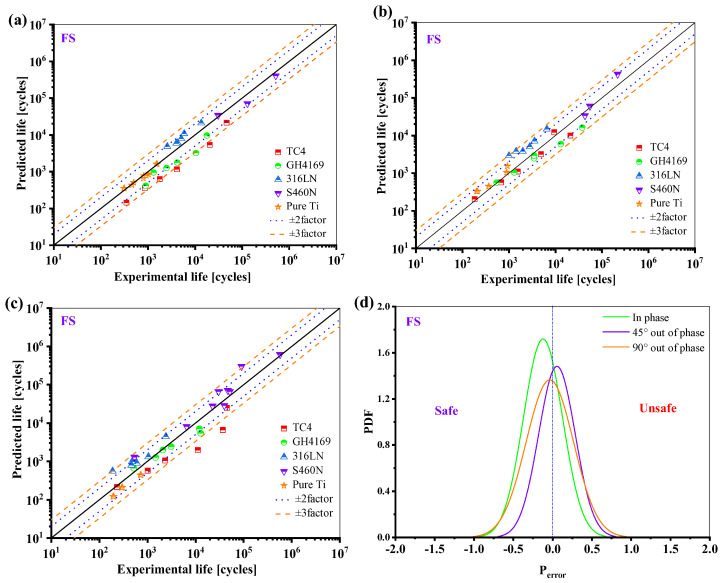
Prediction results of FS model: (**a**) in phase; (**b**) 45° out of phase; (**c**) 90° out of phase, and (**d**) the probability density function of the *P*_error_.

Most of the life prediction results of the FS model for the five materials are in the range of ±3 times the scattering band, and it can be seen from Figure 18d that the life prediction results of this model are more conservative and accurate under proportional loading.

**Figure 19:**
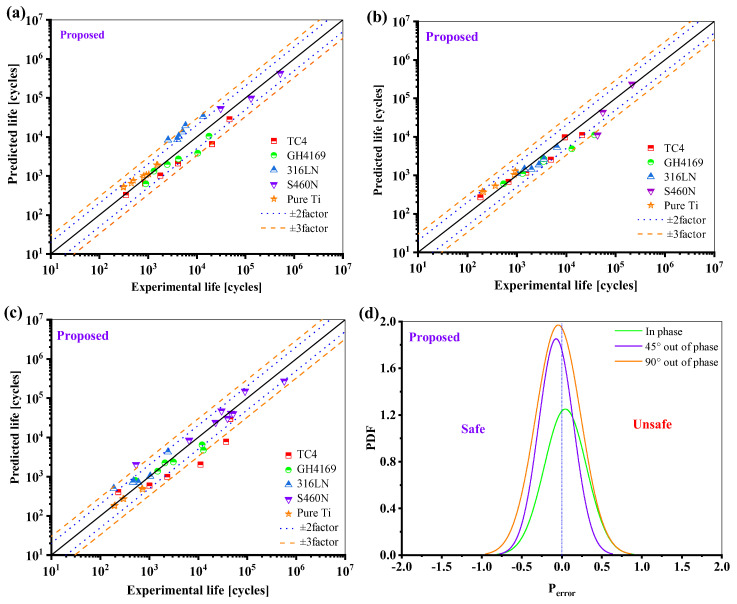
Prediction results of the proposed model: (**a**) in phase; (**b**) 45° out of phase, (**c**) 90° out of phase, and (**d**) the probability density function of the *P*_error_.

Figure 19 shows that for both proportional and non-proportional load paths, most of the prediction results of the proposed model for the five materials fall within the range of ±3 times the scattering band. It is critical to note that the ductility adjustment function has improved the model’s life prediction results under non-proportional loadings, as seen in Figure 19d; the new model is safer, less scattered, and more accurate for life under non-proportional loads.

**Figure 20:**
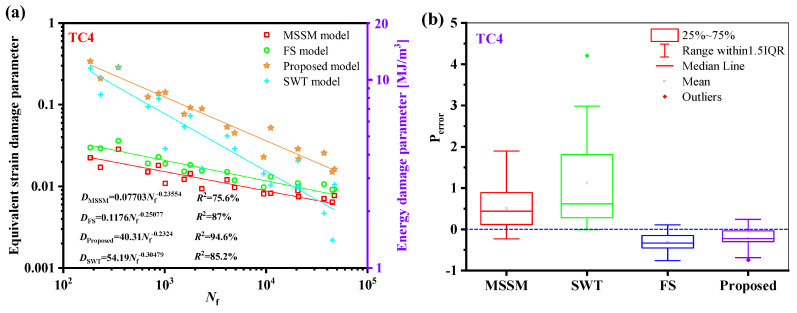
(**a**) Damage parameters vs. fatigue life and (**b**) box plot of prediction errors for TC4. under multiaxial fatigue loadings.

**Figure 21:**
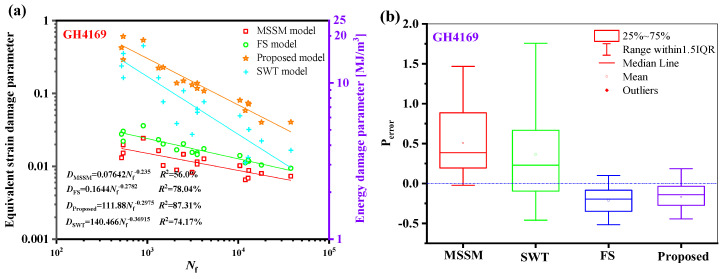
(**a**) Damage parameters vs. fatigue life and (**b**) box plot of prediction errors for GH4169 under multiaxial fatigue loadings.

Regarding the TC4 prediction outcomes, as illustrated in Figure 20b, the new model outperforms the other three models. Comparably, Figure 21a shows that the new model’s damage parameters are less scattered regarding GH4169. Figure 21b shows that the new model’s prediction results are more concentrated and that the mean value is closer to 0.

**Figure 22:**
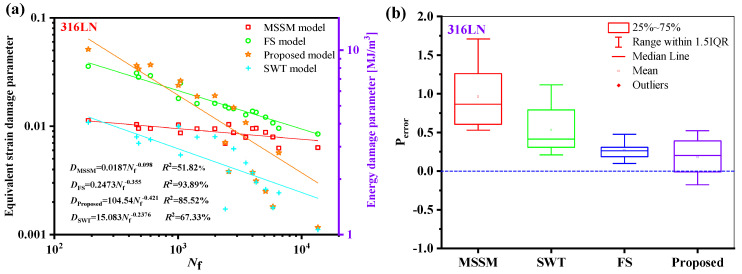
(**a**) Damage parameters vs. fatigue life and (**b**) box plot of prediction errors for 316LN under multiaxial fatigue loadings.

Figure 22a illustrates that, for the 316LN high ductility material, the dispersion of the new model is superior to the FS model but not as excellent as the MSSM and SWT models. The life prediction findings of 316LN are overestimated by the SWT and FS models. Because the ductility adjustment function has been considered, the new model can effectively increase the damage value and decrease the predicted life, causing the prediction results to be spread on both sides of the experimental value. However, compared to the prediction outcomes of the FS model, the new model is less concentrated.

**Figure 23:**
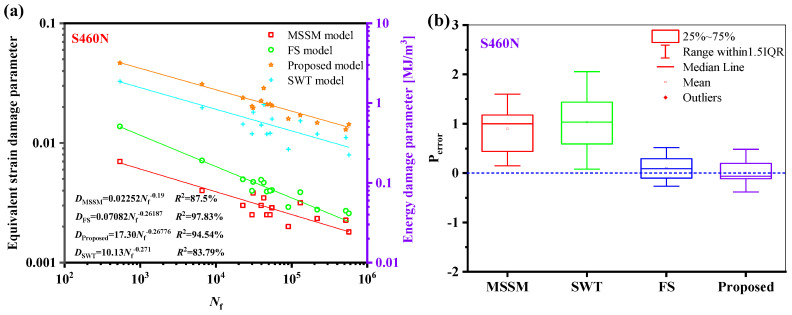
(**a**) Damage parameters vs. fatigue life and (**b**) box plot of prediction errors for S460N under multiaxial fatigue loadings.

**Figure 24:**
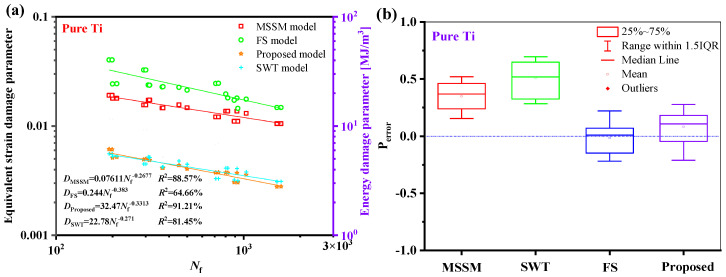
(**a**) Damage parameters vs. fatigue life and (**b**) box plot of prediction errors for pure Ti under multiaxial fatigue loadings.

The suggested damage parameters can be concentrated in a narrow dispersion zone, as seen in Figure 23a and Figure 24a. As shown in Figure 23b and Figure 24b, the fatigue life prediction results of the new model for S460N and pure Ti are more accurate than those of the MSSM and the SWT models. However, the life prediction results of the new model for S460N and pure Ti are similar to those of the FS model.

**Figure 25:**
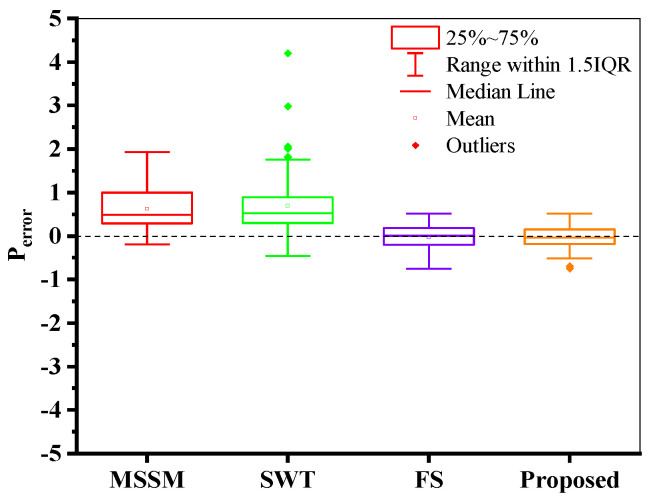
Box plot of four models’ prediction errors for five checked materials.

As shown in Figure 25, the prediction results of the four models are distributed on both sides of the experimental values. Still, the MSSM and the SWT model both give excessively high life prediction results, and the prediction ability of the FS model and the proposed model is similar. It is evident from the fatigue life prediction results of the five materials mentioned above that the new model’s results are reliable and accurate.

Based on the above studies, it is reasonable to consider the ductility parameter of the material as a non-proportional load sensitive parameter under multiaxial loads; however, the model was proposed based on macroscopic phenomenology, and the influence mechanism of ductility on fatigue life should be studies in the future.

## 6. Conclusions

In this paper, by taking into account the differences in the material ductility under non-proportional loading, as well as the mutual promotion of shear stress and normal stress on the crack surface, a new multiaxial fatigue prediction model is established. Test data from six materials were chosen to confirm the reliability and accuracy of the proposed model. The practical results show the following:

(1) Practice demonstrates that the new damage parameter satisfies the following presumptions: (1) The plane exhibiting the maximum shear strain range is the critical plane of a material with shear failure characteristics. (2) The greater the ductility, the more sensitive the material is to an out-of-phase load, the greater the damage parameter, and the shorter the fatigue life. (3) The normal work and shear work on the critical plane leading to fatigue failure do not have the same weights. (4) The shear component and the normal component on the crack surface have mutually reinforcing effects.
(2) Most of the lifetime prediction results of the proposed model for AA2024-T351 are within the ±2 times scattering band.
(3) The fatigue life predictions of the proposed model for five materials in the literature under multiaxial loading fall within the range of ±3 times the scattering band. In contrast to the model without a ductility adjustment function, the prediction results of the new model are less scattered and safer, and more accurate.
(4) Compared with the MSSM and SWT models, the proposed model can give more accurate and safer prediction results for the fatigue life under both proportional and non-proportional loads. In contrast to the popular FS model, the new model performs better life prediction under non-proportional loading.

## Figures and Tables

**Table 2:** Mechanical properties of AA2024-T351.

*σ*_y_ [MPa]	*σ*_u_ [MPa]	*E* [GPa]	*H_v_* [HV]	*δ* [%]	*G* [GPa]
300.1	460	73	137	10	27

**Table 3:** Fatigue parameters of AA2024-T351.

$\sigma_{f}^{\prime }$ [MPa]	$\varepsilon_{f}^{\prime }$ [−]	*b* [−]	*c* [−]
1066.3	0.2332	−0.14	−0.66

**Table 4:** Multiaxial fatigue life of AA2024-T351 under tensile–torsional loading [28].

No.	Strain Path	Δε_eq_/2 [%]	Δε/2 [%]	Δγ/2 [%]	Strain Amplitude Ratio	*N*_f_ [Cycles]	Mean Value [Cycles]
N1	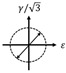	0.55	0.391	0.676	1.73	7709	7229
N2						5530	
N3						8448	
N4			0.45	0.56	1.24	6905	6905
N5	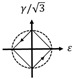	0.55	0.391	0.676	1.73	11,107	11,191
N6						12,854	
N7						9613	
N8			0.45	0.56	1.24	12,712	12,175
N9						11,637	

**Table 5:** Uniaxial fatigue parameters of the checked materials.

Materials	TC4	GH4169	316LN	S460N	Pure Ti
Refs.	[12]	[34]	[46]	[47]	[48]
*E* [GPa]	108.4	198.5	190	208.5	112
*G* [GPa]	43.2	67	79	80.2	40
$\gamma_{f}^{\prime }$ [MPa]	2.24	4.46	0.766	-	0.417
$\tau_{f}^{\prime }$ [MPa]	716.9	1091.6	688	-	485
*b*_0_ [−]	−0.06	−0.07	−0.135	-	−0.069
c_0_ [−]	−0.8	−0.77	−0.451	-	−0.523
$\varepsilon_{f}^{\prime }$ [−]	0.579	0.45	-	0.281	0.548
$\sigma_{f}^{\prime }$ [MPa]	1116.9	1815.5	-	969.6	647
*b* [−]	−0.049	−0.06	-	−0.086	−0.033
*c* [−]	−0.679	−0.63	-	−0.493	−0.646
*v_e_* [−]	0.25	0.48	0.3	0.3	0.4
$\sigma_{y}$ [MPa]	942.5	1083.1	292	500	475
Elongation [*%*]	18.3 [28]	26 [49]	81 [50]	26.2 [51]	17.5 [52]

## Data Availability

The raw data supporting the conclusions of this article will be made available by the authors on request. As the raw data for AA2024-T351 in the manuscript is part of an ongoing study, further details on AA2024-T351 cannot be provided. Therefore, the data provided is limited.

## Nomenclature

Δ*γ*_max_	the maximum shear strain range acting on the critical plane
*σ*_n,max_	maximum normal stress acting on the critical plane
Δ*τ*_t_	shear stress acting on the critical plane
Δ*σ*_n_	normal stress acting on the critical plane
Δ*ε*_n_	normal strain acting on the critical plane
*k*	material constant of FS model
*σ*_y_	the yield strength
*σ_u_*	the ultimate strength
*H_v_*	Vickers hardness
*φ*	phase difference
v	Poisson’s ratio
$\tau_{f}^{\prime }$	shear fatigue strength coefficient
$\gamma_{f}^{\prime }$	shear fatigue ductility coefficient
*b*_0_	shear fatigue strength exponent
*c*_0_	shear fatigue ductility exponent
$\sigma_{f}^{\prime }$	fatigue strength coefficient
$\varepsilon_{f}^{\prime }$	fatigue ductility coefficient
*b*	fatigue strength exponent
*c*	fatigue ductility exponent
*E*	modulus of elasticity
*G*	shear modulus
*N*_ipf_	fatigue life under proportional load
*N*_f_	fatigue life
*N*_nonpf_	fatigue life under non-proportional load
*N*_d_	the degree of life reduction due to non-proportional loading
*Ψ*	load non-proportional factor
*δ*	elongation
Δ*γ*_e_/2	elastic strain amplitude
Δ*γ*_p_/2	plastic strain amplitude
Δ*γ*/2	total strain amplitude
*P*_error_	life prediction error
*N*_fp_	predicted life
*N*_ft_	actual life
